# The Role of Sphingolipids in Cancer Immunotherapy

**DOI:** 10.3390/ijms22126492

**Published:** 2021-06-17

**Authors:** Paola Giussani, Alessandro Prinetti, Cristina Tringali

**Affiliations:** Department of Medical Biotechnology and Translational Medicine, Università degli Studi di Milano, LITA Segrate, Via Fratelli Cervi, 93, 20090 Segrate, Italy; paola.giussani@unimi.it (P.G.); alessandro.prinetti@unimi.it (A.P.)

**Keywords:** sphingolipids, sphingosine 1-phosphate, immunotherapy, cancer

## Abstract

Immunotherapy is now considered an innovative and strong strategy to beat metastatic, drug-resistant, or relapsing tumours. It is based on the manipulation of several mechanisms involved in the complex interplay between cancer cells and immune system that culminates in a form of immune-tolerance of tumour cells, favouring their expansion. Current immunotherapies are devoted enforcing the immune response against cancer cells and are represented by approaches employing vaccines, monoclonal antibodies, interleukins, checkpoint inhibitors, and chimeric antigen receptor (CAR)-T cells. Despite the undoubted potency of these treatments in some malignancies, many issues are being investigated to amplify the potential of application and to avoid side effects. In this review, we discuss how sphingolipids are involved in interactions between cancer cells and the immune system and how knowledge in this topic could be employed to enhance the efficacy of different immunotherapy approaches. In particular, we explore the following aspects: how sphingolipids are pivotal components of plasma membranes and could modulate the functionality of surface receptors expressed also by immune cells and thus their functionality; how sphingolipids are related to the release of bioactive mediators, sphingosine 1-phosphate, and ceramide that could significantly affect lymphocyte egress and migration toward the tumour milieu, in addition regulating key pathways needed to activate immune cells; given the renowned capability of altering sphingolipid expression and metabolism shown by cancer cells, how it is possible to employ sphingolipids as antigen targets.

## 1. Introduction

The establishing of complex and particular interactions between tumour cells and immune system occurs during tumorigenesis and facilitates the expansion of transformed cell clones. The immune system is usually able to counteract growing tumour cells, as demonstrated by the evidence that immunocompromised animal models or human immunodeficiency virus (HIV) infected humans are more susceptible to cancer. On the other hand, tumour cells can acquire the capability to escape this control, by favouring the formation of an immunosuppressive microenvironment. For the past 15 years, the dynamic interplay between tumours and immune system has been conceptualised by a theory called “Immunoediting,” reviewed by Muenst [1]. Three interrelated phases are theorized: (a) elimination, i.e., the attempt of innate and adaptive immunity for eradicating tumour cells; (b) equilibrium that is a sort of silent coexistence of the immune system and tumour cells; and (c) escape when tumour cells finally conquer the final battle with the immune system and can grow and spread unconditionally. The success of the tumour is mediated by different tools, mainly, on one hand, the impaired ability of immune cells to recognize tumour cells and, on the other, the increased survival of tumour cells and their capacity even to gain a benefit from some immune responses such as inflammation. Different immune players are involved in the fight against cancer: firstly, antigen presenting cells (APCs) retain and present tumour antigens to T cell effectors, activating them to kill tumour cells [2]. Furthermore, CD8^+^ memory T cells are able to act against tumour cells. On the other hand, regulatory T cells (Tregs), which are responsible for self-tolerance, could block CD8^+^ T and B cells, APCs, and natural killers (NKs) and are largely enticed by chemokines released by tumour cells and macrophages [3]. NKs recognize tumour cells through different membrane receptors, such as the activating NK receptor, NKG2D [1,4,5]. Tumour-associated macrophages (TAMs) are actively recruited in the tumour microenvironment and play a dual role: M1 macrophages releasing Th1 cytokines, including interleukin (IL)12 and tumour necrosis factor (TNF)α, block cancer cells, while M2 macrophages, releasing Th2 cytokines such as IL6, IL10, and transforming growth factor (TGF)β, favour cancer expansion, promoting angiogenesis and inhibiting T cell activation [1,6]. Similarly, tumour-associated neutrophils (TANs) can both suppress and favour tumour progression. In particular, in response to different signals released by the tumour microenvironment, such as TGFβ, they shift toward a pro-tumour phenotype (N2 TANs), whereas interferon (IFN)β stimulates the neutrophil anti-tumour phenotype (N1) [7].

Improving the immune response toward tumours is now considered a strong and promising weapon to eradicate cancer, in particular those types resistant to conventional therapies or largely diffuse. Immunotherapy is performed following two strategies: the first, known as passive immunotherapy, is represented by the employment of monoclonal antibodies, adoptive cell therapy, and chimeric antigen receptor T (CAR-T) cells; the second, i.e., the active immunotherapy, strengthens the host immune response, administrating vaccines, cytokines, or checkpoint inhibitors [8,9].

In this context, current knowledge regarding sphingolipids could play a significant role. Sphingolipids arise from a common molecular core constituted by ceramide (Cer) that is, in turn, formed by a fatty acid linked to sphingosine. The addition of a residue of phosphocoline to the 1-hydroxyl group of Cer gives rise to sphingomyelin. Instead, when one sugar or a carbohydrate chain is linked to the same position, glycosphingolipids are originated. Specifically, glycosphingolipids are named: (a) cerebrosides, when galactose or glucose residues are linked to ceramide; (b) sulphatides, when cerebrosides are sulphated; (c) gangliosides, if the oligosaccharide chain includes one or more sialic acid residues. The synthesis and release of sphingosine 1-phosphate (S1P) is directly interrelated to sphingolipid metabolism. The chemical structures of the sphingoid and sphingolipid molecules cited in this review are described in Table 1. Sphingolipid expression and S1P release are crucially compromised in cancer, leading to the appearance of many tumour-associated antigens and to deregulated signalling [10,11,12]. Tumour sphingolipids originate from neo-synthetic processes, over-expression of some species such as gangliosides or aberrant sialylation due to *O*-acetylation of sialic acid or to the acquired capability to incorporate the unusual form *N*-glycolylneuraminic acid (Neu5Gc) of sialic acid instead of *N*-acetylneuraminic acid (Neu5Ac) [13]. Significantly, humans cannot synthesise Neu5Gc, which is mainly derived from diet and is retained at very low levels in healthy tissues [14].

Different aspects related to sphingolipid biology and metabolism can be exploited in immunotherapy. First, glycosphingolipids are able to form functional clusters on the plasma membranes, in association with receptors, adhesion molecules, and signalling players, which can interfere with immune cell activation. On the other hand, bioactive sphingoid molecules such as S1P and Cer can alter intracellular signalling pathways related to immune cell activation or survival. Therefore, these aspects could be considered to enhance immune response. Finally, sphingolipid antigens can be employed as targets for different immunotherapies.

In this review, we will explore how data concerning sphingolipids and sphingoid molecules in tumours and immunity can benefit cancer immunotherapy, and which strategies have been by now attempted and established.

## 2. The Multifaceted Functions of Sphingolipids in the Interplay between Cancer Cells and Immune System

A long time before the discovery of the functional roles of the so-called “bioactive sphingolipids”, Cer and S1P, several groups, in particular the one led by late Sen-itiroh Hakomori, discovered deep anomalies in the metabolism and expression of glycosphingolipids, in particular gangliosides, in several types of tumours [15,16,17]. Aberrant glycosphingolipid expression in tumour tissues is indeed a typical aspect of a more general phenomenon typical of tumour cells, “aberrant glycosylation”, i.e., the expression of carbohydrate epitopes in glycolipids, glycoproteins, and glycosaminoglycans at levels or with structures different than in normal tissues or cells. In the case of glycolipids, very heterogeneous situations have been described. In some tumours, the total ganglioside levels were higher than in the corresponding healthy tissue (e.g., breast tumour tissues versus normal mammary tissues [18]). As mentioned above, gangliosides in some tumours do contain sialic acids with anomalous structures. Frequently, specific glycosphingolipids do accumulate in specific types of cancer: a few relevant examples include GD3, GD2 and GM3 in melanoma [19], GD2 in neuroblastoma, Gg3 in lymphoma [19], fucosyl-GM1 in small cell lung carcinoma, and Globo-H in breast and ovarian carcinoma (reviewed in [16]). In some cases, tumours or tumour cell lines of similar origins, but with different phenotypes in terms of aggressiveness/invasiveness/metastatic potential (and prognosis, in the case of human cancers), were characterized by very different levels of the same glycosphingolipid. The best-studied example is probably represented by GM3 ganglioside in bladder [20,21] and ovarian cancer [22,23], where an inverse correlation has been observed between GM3 expression and invasive potential of the tumour cells.

These studies strongly drove research that has revealed the role of glycosphingolipids in regulating properties of the tumour cells and the potential for tumour-specific glycolipid antigens to serve as targets for different kinds of immunotherapy, as detailed in the next section.

In addition, several studies demonstrated that cultured tumour cells released significant amounts of selected ganglioside molecular species in the extracellular milieu [24,25], and that serum and cerebrospinal fluid levels of gangliosides were higher in cancer patients [26,27]. This, together with the observation that exogenous gangliosides might exert modulatory effects on some populations of immune cells [25], suggested that the release of gangliosides by tumours could be part of an immune escape or immune-reprogramming strategy. However, the molecular basis of the effects of tumour-released gangliosides remained elusive, and this knowledge has not led to the development of effective strategies. Only recently it has been demonstrated that circulating very long chain fatty acid (22:0, 23:0, and 24:0) containing GM3 species do affect the activation of Toll-like receptors, whose prolonged and abnormal activation is one of the factors leading to low-grade chronic inflammation, a common trait of different disorders including insulin resistance and cancer [28]. This suggests that tumour-released gangliosides might have a significant role in the process of meta-inflammation and in reprogramming cellular populations involved in the innate immune response.

As explained above, several lines of evidence indicated the importance of glycosphingolipids in regulating cellular functions such as proliferation, adhesion, and motility, and the relevance of dysregulation of these events due to aberrant glycosphingolipid expression for the tumour phenotype [29,30]. Glycosphingolipids, and in particular gangliosides, are highly enriched in plasma membranes, and their cellular actions are mainly due to their capability to interact with plasma membrane receptors (including growth factor tyrosine kinase receptors, G-coupled receptors, and adhesion receptors such as integrins) modulating their functional activity [31]. In some cases (e.g., the modulation of epidermal growth factor receptor (EGFR) activity with consequent inhibition of cellular proliferation by GM3 [32]), the effect is due to a direct lateral glycolipid-receptor interaction. However, spontaneous segregation of some membrane lipids in platforms characterized by a liquid-ordered phase-like behaviour leads to the formation of specialized membrane domains, the lipid rafts. Lipid rafts are enriched in gangliosides, sphingomyelin, cholesterol, and several classes of selected proteins involved in signal transduction. The co-clustering of different molecules in glycolipid-enriched areas organizes and determines the function of multiprotein complexes involved in several aspects of signal transduction, thus regulating cell homeostasis, even in the absence of direct specific glycolipid-protein interactions [33,34]. Obviously, the aberrant glycolipid composition of tumour cells implies the formation of anomalous lipid rafts, and alterations in raft-associated signalling in tumour cells have been extensively documented [29].

However, even if much less studied, immune cells functions are at least in part due to the organization of specific lipid rafts at their surface. The best-studied example at the cellular and molecular level is represented by neutrophils, whose ability to effectively recognize, phagocytose, and kill microorganisms is linked to specialized lactosylceramide-enriched lipid rafts [35,36,37]. This example is not directly relevant to the interplay between tumours and the immune system, while several lines of evidence suggest the importance of the association with lipid rafts for several immune system cell receptors, including T- [38,39] and B-cell [40,41] receptors in lymphocytes, and CD1b in dendritic cells/monocytes [42]. Partitioning into lipid rafts seems to be relevant not only for signalling, but also for trafficking of these receptors [43], and proteins controlling the association of receptors with lipid rafts, such as the caspase-recruitment domain (CARD)-membrane-associated guanylate kinase (MAGUK) protein 1 (CARMA1), have been suggested as potential therapeutic targets [44]. Moreover, the simple sphingoid mediator S1P, able to exert its effects on many different cell types via a family of specific membrane G-coupled membrane receptors, plays crucial roles in the control of the activation and migration of different immune cell populations (as discussed in detail below). Remarkably, not only S1P receptors, but also the main enzymes involved in the control of S1P levels seem to be localized into lipid rafts (reviewed in [45]).

## 3. Targeting S1P Signalling to Improve Immunotherapy

Different sphingolipids have emerged as mediators involved in the regulation and control of inflammatory pathological conditions including cancer, inflammatory bowel disease (Crohn’s disease and ulcerative colitis), atherosclerosis, type II diabetes, rheumatoid arthritis, and obesity. In particular, bioactive sphingolipids such S1P and Cer act as second messengers and have a critical role in the regulation of cell proliferation and fate [46,47]. Cers promote cell cycle arrest and apoptosis, and several pieces of evidence supported the theory that S1P exerts a critical role in cancer progression and treatment [48,49,50,51,52]. S1P, which is synthetized by sphingosine kinases (SKs), reduces apoptosis and enhances cell growth. Accordingly, inflammatory aspects related to cancer spread and progression involve SKs [53].

**Figure 1:**
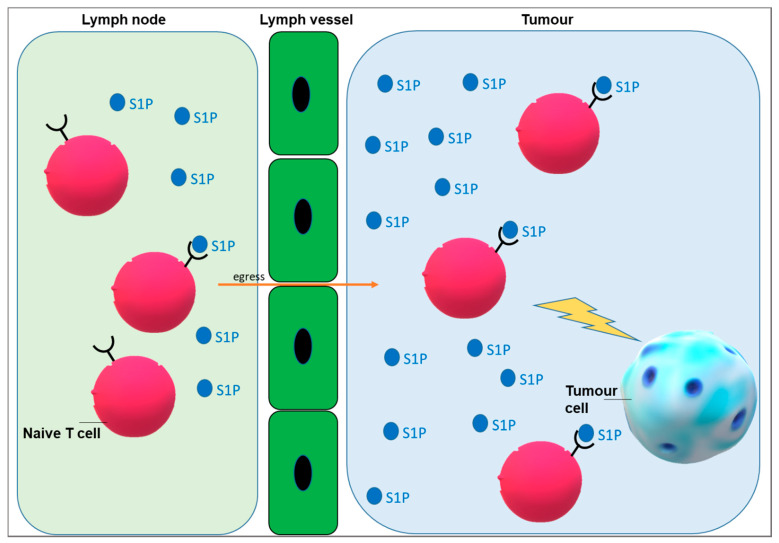
**Model of the role of S1P (sphingosine 1-phosphate) in T cell egress and migration in tumour milieu.** T cells express surface S1PR1 (sphingosine 1 phosphate receptor 1) when they are inside the lymphoid tissue. S1PR1-mediated S1P signalling in T cells is needed for the exit from the lymphoid tissue.

Thus, alterations related to S1P and Cer release could have deep consequences on immune system response against cancer and on immunotherapy efficacy. First, the enhanced synthesis of Cer, in addition to S1P and glucosylceramide, is a tool employed by cancer cells to increase survival [46]. Short chain Cers such as C2 Cer and C6 Cer have been demonstrated to be involved in the modulation of immunocytes and in the proinflammatory responses [54,55]. Moreover, in inflammatory diseases, sphingolipids are able to act as anti- or pro-inflammatory mediators and participate in controlling leukocyte activation and migration. It has been demonstrated that S1P metabolism and signalling are often impaired during infections [56]. To increase the efficacy of cancer immunotherapy, it is useful to target sphingolipid metabolism and signalling, mainly S1P which has been demonstrated involved in lymphocyte egress (Figure 1) [57].

Apoptotic cells are physiologically removed by efferocytosis and secrete chemoattractant factors and endogenous proliferative factors that warn the adjacent cells and tissues for regeneration and clearance [58]. During this process, apoptotic cells release intracellular constituents such as lysophosphatidylcholine (LPC) [59,60] that act as short-range chemo-attractants recruiting immune cells as well as S1P [61] processed by the caspase-dependent activation of SK1/SK2 [62]. Chemo-attractants and other soluble factors released from apoptotic cells induce migration and chemo-attraction of immune cells (mostly myeloid-derived phagocytes such as macrophages and dendritic cells) [58,59,60,61]. Extracellular vesicles exosomes, microvesicles, apoptotic bodies, and extracellular membrane-delimited particles that are secreted by apoptotic cells can influence indirectly the local microenvironment. Extracellular vesicles from apoptotic cells contain nucleic acids proteins, and lipids able to induce proliferation of neighbouring cells and function as a chemoattractant in the tissue microenvironment [58]. Moreover, a number of anti-inflammatory factors are produced by phagocytic cells during efferocytosis; together with apoptosis, these events participate in the homeostasis that regulates tissue repair [58]. The processes mentioned above, if not well regulated, can open to cancer progression and immune escape [58].

It is known that in blood and lymph flow there are high levels of S1P, but its levels in tissues are low. S1P gradients are fundamental for lymphocyte egress and other aspects of physiological cell trafficking (Figure 1). S1P lyase, responsible of irreversible S1P degradation, regulates different physiological and pathological processes such as lymphocyte trafficking and inflammation [63,64,65,66,67]. For example, Kumar and colleagues demonstrated that S1P lyase located in thymic dendritic cells controls the normal egress of T lymphocytes from the thymus into the circulation. Instead, S1P lyase deficiency in gut epithelial cells induces colitis and colitis-related tumorigenesis [65].

It has been shown that S1P regulates lymphocyte egress into circulation via S1P receptor 1 (S1PR1) signalling, and it controls the differentiation of Tregs and T helper-17 cells [68]. Liu and collaborators demonstrated that Akt-mTOR pathway activation by S1P/S1PR1 inhibits the development and function of Tregs [69]. Thus far, the S1P pro-survival role is well studied by the scientific community, but the role of S1P in modulating T cell metabolism for controlling anti-tumour immune response is not well known. Moreover, not only extracellular S1P, but also intracellular S1P regulates different signal transduction pathways binding directly to its targets [68,70,71,72,73,74]. However, it is not fully known how receptor-independent intracellular S1P levels modulate T cell functionality [68]. Chakraborty and collaborators demonstrated that S1P generated by SK1 and the activity of the lipid transcription factor peroxisome proliferator-activated receptor (PPAR)γ, which in turn regulates lipolysis in T cells, are directly correlated. Inhibition of SK1 improved anti-tumour activity of T cells against murine melanoma suggesting the clinical potential of the limitation of the signalling SK1/S1P-mediated to promote the anti-tumour T cell therapy [68].

Compounds able to target S1P signalling pathways are of interest for immune system regulation. It has been demonstrated that S1P signalling controls many more cell types and processes than previously indicated [57,75].

On the other side, cancer stem cells (CSCs) are resistant to the chemotherapeutic drugs and it has been recently demonstrated that to have a successful cancer therapy it is important to target immunologically CSCs [76,77,78]. On these bases, it is very important to specifically target the CSCs for an effective cancer immunotherapy. Moreover, it is known from the literature that in CSCs sphingolipid metabolism is dysregulated [51,78]. High levels of Cer induce cell death in cancer cells [47,79,80,81], but also enhance host protective immune response inducing cancer regression [80,81]. Moreover, it has been demonstrated that CSCs are characterized by high levels of S1P [51]. Augmented S1P has been demonstrated to (i) induce the increase of the anti-apoptotic proteins Bcl-2 and Bcl-XL in macrophages and corresponding M2 type polarization [82,83,84] and (ii) recruit immune cells to the immune tissues to make the immune response operative. In particular, S1P released by apoptotic or tumour cells binds to its specific receptor (S1PR1) inducing the recruitment of monocytes circulating in the tumour microenvironment [85]. Furthermore, in hypoxic tumour microenvironment, S1P acts as chemoattractant of T cells and NK cells [86,87,88].

## 4. Employment of Glycosphingolipids in Immunotherapy against Cancer

### 4.1. Creating Vaccines against Cancer

Vaccines are believed to elicit a strong host immune cellular response against developing cancer. This type of therapy is fundamentally based on the choice of specific tumour antigens able to efficiently promote T-cell activation, the choice of adjuvants and of the delivery method [89]. Thus far, many obstacles impaired the success of vaccines in cancer therapy such as the action of immunosuppressive Tregs, the high release of immunosuppressive cytokines, such as IL10, TGFβ, IL2, and of T-cell checkpoint ligands in the tumour milieu [89]. Glycosphingolipids were chosen as ideal targets for mounting the immune response because of their altered expression in roughly all tumours, as discussed above.

Nevertheless, additional problematics have emerged soon. First, the choice of glycolipid: some glycosphingolipids are expressed also by healthy tissues different from that from which tumour arises. In addition, they could be tolerated by the immune system and therefore are low immunogenic. To increase immunogenicity, synthetic glycosphingolipids have been linked to keyhole limpet haemocyanin (KLH), bovine serum albumin (BSA), or tetanus toxoid (TT) [13].

**Figure 2:**
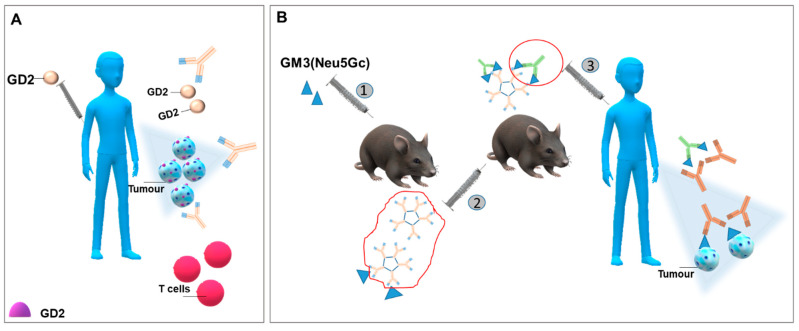
**Gangliosides as antigens for formulating vaccines against tumours.** (**A**) Scheme of a vaccine planned to stimulate a response against ganglioside GD2. GD2 is administered associated to KLH (keyhole limpet haemocyanin), BSA (bovine serum albumin), or TT (tetanus toxoid) and elicits an antibody response against tumour cells exposing the same ganglioside. This, in turn, activates a specific anti-tumour T cell response. (**B**) Schematic picture of Racotumomab (1E10) preparation and action. (1) GM3 (Neu5Gc), enclosed in liposomes, is injected into a Balb/c mouse and induces the release of IgM antibodies; (2) IgM antibodies targeting GM3 (Neu5Gc) are isolated and further injected into a second Balb/c mouse, triggering the release of IgG anti-idiotype antibodies. These last antibodies are known as Racotumomab and (3), when they are injected into a patient, are able to recognize cancer cells expressing GM3 (Neu5Gc) and to stimulate the immune response.

Different gangliosides, i.e., GD2, GD3, GM1, GM2, and GM3 were tested with the aim of creating a vaccine, also after transforming them into lactone structures, as reviewed by Durrant [13], but rarely were these approaches able to trigger an IgG response or to significantly improve patients’ survival [90,91]. Patients with high-risk neuroblastoma in second remission were treated with a bivalent vaccine GD2L-KLH+GD3L-KLH with OPT-821 as adjuvant and oral-barley-derived (1→3), (1→4)-β-D glycan. The treatment gave rise to the strong appearance of anti-GD2 and anti-GD3 IgG antibodies and to long event-free survival (EFS) [92] (Figure 2A). GM3(Neu5Gc) has been considered to develop vaccines due to its rarity in healthy cells. In a multicentre Phase I/III study, GM3(Neu5Gc) enclosed in proteoliposomes was subcutaneously injected in patients affected by metastatic cutaneous melanoma. The vaccination promoted anti-GM3(Neu5Gc) IgM and IgG response [93]. Racotumomab is an anti-idiotype monoclonal antibody (IgG1), formerly known as 1E10, isolated after the administration of GM3(Neu5Gc) to Balb/c mice to produce an IgM monoclonal antibody (mAb1) that has been further injected in mice. Racotumomab specifically recognizes mAb1, mimics GM3(Neu5Gc) and, when injected in patients, stimulates the release of antibodies against GM3(Neu5Gc) [94,95] (Figure 2B). Racotumomab is currently recorded in Cuba and Argentina as second line treatment for non-small lung cancers (NSCLC) and is being evaluated also for melanoma, breast cancer, and neuroblastoma [96]. The employment of this vaccine has been correlated to survival in patients affected by different types of lung cancers, along to the expression of GM3(Neu5Gc) in malignant tissues [97]. 1E10 vaccine employed in patients with stage III and IV breast cancer induced specific antibody release and T-cell response in about half of subjects [98]. Further, anti-GM3 (Neu5Gc) vaccine appeared to be able to strengthen the anti-metastatic effects of anti-EGFR mAbs (7A7 mAb) [99].

Recent evidence suggested also the employment of sphingolipids, i.e., galactosylceramide, as immune adjuvants in vaccines formulated against other tumour antigens and demonstrated a potential efficacy of this approach [100]. In addition, ganglioside-liposomes have been planned to convey antigens specifically to APCs [101].

### 4.2. Generating Antibodies against Glycosphingolipids Expressed by Cancer Cells

**Figure 3:**
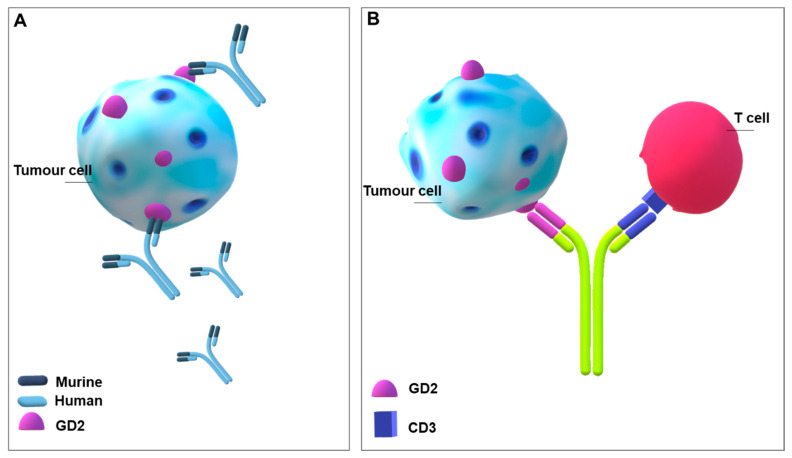
**Gangliosides as antigens for building monoclonal antibodies against tumour cells.** (**A**) Picture of the monoclonal antibody Ch14.18 mAb (dinutuximab). (**B**) Illustration of a bispecific antibody targeting tumour-associated GD2 and CD3 on a T cell.

Monoclonal antibodies (mAb) generated toward glycosphingolipids promote cell death through oncosis, antibody-mediated cellular cytotoxicity (ADCC), or complement-dependent cytotoxicity (CDC) [13]. mAbs were produced toward GD2 (14.G2a, ch14.18, ch.60C3, 3F8, KM8138), GD3 (MB3^.^6, R24), GM2 (DMF10^.^167^.^4), and GM3 (Neu5Gc) (14F7) [13]. Ganglioside GD2 is the main target considered for immunologic treatment of neuroblastoma [102] and has been ranked 12th among the main tumour-related antigens by the National Cancer Institute [103]. Ch14.18 mAb (dinutuximab) was approved by Food & Drug Administration (FDA) and European Medicines Agency (EMA) for high-risk neuroblastoma patients in combination with other therapies, and increases five-year survival by 20% [104]. The same antibody has been demonstrated to be efficacious against triple negative breast cancer [105]. Ch14.18 Ab is a human-murine chimeric antibody obtained by joining the variable region of murine IgG3 anti-GD2 mAb 14.18 and the constant region of human IgG1 [92,105] (Figure 3A). Ch14.18 was then produced in Chinese hamster ovary (CHO) cells instead of the murine myeloma cell line, SP2/0, giving the mAb known as dinutuximab-beta with similar GD2 binding, toxicity, and pharmacokinetics [92]. The therapy has considerable significant side effects, including neuropathic pain. Therefore, other approaches were considered to avoid these complications.

Humanized antibodies Hu3F8 [106] and Hu14.18K322A [107,108] were generated to this aim and their efficacy and side effects are being investigated in different clinical trials for resistant, recurrent, or high-risk neuroblastomas.

Concerning GD2, bispecific antibodies have also been engineered; bispecific antibodies are designed to attract T cells in the tumour environment. In this case, they are able to recognize GD2 with one arm and to attract cytotoxic lymphocytes with the other, recognizing CD3 molecules expressed by T cells [102,109] (Figure 3B). Surek is a trifunctional bispecific antibody endowed with a higher efficacy than GD2 monospecific antibody. Surek has been tested in C57BL/6J mice inoculated with GD2 positive melanoma cells. It activated both CD4^+^ and CD8^+^ T lymphocytes and Th1 immune response and assured a memory response active also against a next injection of GD2 negative B16 melanoma cells [110]. Along this line, Hu3F8-single-chain variable fragment based bispecific antibody (scBA) stimulated a strong T cell response and significantly prolonged the survival of both melanoma and neuroblastoma xenografts [111]. Thus, based on all data, bispecific antibodies should be more efficacious than conventional anti-GD2 antibodies.

Another strategy pursued to increase the immunogenicity of gangliosides was to consider their *O*-acetylated form. *O*-acetylation, mostly occurring on the C9 of sialic acid residues carried by gangliosides, is a frequent modification shown by tumours of neuroectodermic origin. *O*-acetylated ganglioside species are typical of developmental processes and are quite rarely expressed by healthy tissues, with the exception of skin and brain. Thus, 9-*O*-Ac-GD3 is considered a marker of neuroblastoma, melanoma, and breast cancer, whereas acetylated GD2 has been revealed in neuroblastoma, glioblastoma, lung tumours, etc. [112]. 8B6 monoclonal antibody targeted *O*-acetylated GD2 has been demonstrated to reduce glioblastoma cell growth in vitro and in vivo [113] and to increase temozolomide efficacy toward glioblastoma stem cells [114].

### 4.3. Improving CAR-T Cell Therapy in Solid Tumours: Glycosphingolipids as Targets

CAR-T-cell therapy consists of withdrawal and isolation of patient’s T cells from leukapheresis. T cells are genetically manipulated in vitro in order to express a CAR targeting a specific tumour antigen. After 3–4 weeks, CAR-T cells, equipped to attack and destroy tumour cells, are infused back into the patient’s blood. CAR-T cell therapy is strongly efficacious in patients affected by relapsing tumours resistant to other therapies. Particularly, it has been approved for the treatment of refractory or relapsing diffuse large B-lymphomas including those positive for Epstein-Barr virus (EBV), acute lymphoblastic leukaemia, and primary mediastinal B-cell lymphoma [115]. CAR is constituted by an extracellular motif which binds an antigen, such as CD19 for CD19-positive B-cell-malignancies. The extracellular component is connected to one or more intracellular signalling domains activating T cells (CD3ζ alone in CAR-T of 1st generation; one co-stimulatory domain such as 4-1BB plus CD3ζ in CAR-T of 2nd generation; multiple co-stimulatory domains such as 4-1BB and CD28, plus CD3ζ in CAR-T of 3rd generation). The antigen-binding single chain variable fragment (scFv) binds antigens with higher affinity than T cell receptor (TCR) and does not need antigen processing and presentation [115].

**Figure 4:**
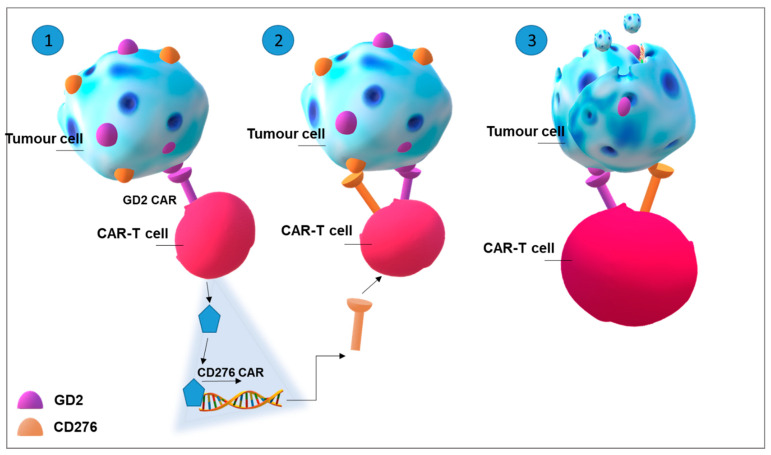
**SynNotch gated CAR-T targeting GD2 (the gate) and CD276 (target) in tumour cells.** Sequence of the events leading to CAR-T cell activation and tumour cell killing. (1) After the binding of the first CAR to GD2, the SynNotch site is cleaved and leads to the release of a transcriptional activator that (2) triggers, specifically the expression of a second gated CAR, recognizing CD276 antigen. Cytotoxic action of T cells is mediated by the expression of the gated CAR. (3) After CD276 binding, CAR-T cell is fully activated and kills the tumour cell.

Despite the successful employment of CAR-T cell therapy in B-cell malignancies, the attempts to apply this therapy in solid tumours have not been as efficacious. This can be primarily due to the identification of specific tumour antigens. In fact, the expression of the same molecules in healthy tissues, even if at very low levels, could give rise to dangerous autoimmune effects [116]. Therefore, the challenges are primarily two: to choose the best antigenic targets and to avoid side toxicity. Due to their aberrant expression in solid tumours, gangliosides are being strongly considered as antigens for CAR-T-therapy [117]. A proposal to overcome the second problematic was to plan a CAR for a tumour antigen that is expressed after a transactivation signal triggered by a second antigen. This strategy is achieved by linking scFv binding a chosen antigen (gating antigen) to a synthetic (Syn) Notch receptor. After the binding of the antigen with scFv, SynNotch receptor activates a transcriptional activator that triggers the expression of a second CAR recognizing a second antigen [118] (Figure 4).

GD2 ganglioside has been suggested as a target for CAR-T cell therapy in metastatic retinoblastoma because of the capability of GD2-CAR-T cells for killing retinoblastoma cells, in vitro [119]. GD2-CAR-T cells were also effective to suppress H3-K27M^+^ diffuse midline glioma cells [120], neuroblastoma cells in orthotopic xenograft models [121], Ewing sarcoma cell growth and metastatic spread [122], and osteosarcoma cell growth [123]. First generation GD2-CART-T cell therapy has been applied in a phase 1 clinical study in patients affected by relapsing/refractory neuroblastoma and showed that the treatment has been well tolerated even if the efficacy should be improved [124,125]. A phase 1 study enrolling three consecutive relapsed or refractory neuroblastoma patients has been performed employing GD2-CAR-T cells of 3rd generation carrying both CD28 and OX40 as stimulatory domains. Parallel immunodepletion induced by cyclophosphamide and fludarabine administration enhanced GD2-CAR-T cells expansion. Overall, the treatment has been run without significant manifestations of toxicity, but anti-tumour response after 6 weeks was modest [126].

Unfortunately, GD2-CAR-T cell therapy trials have also been associated with negative effects including a lethal encephalitis in human neuroblastoma xenograft treated with GD2-E101K CAR-T cells, caused by massive destruction of brain neurones induced by CAR-T cells [127]. Aiming at controlling neurotoxicity of GD2-CAR-T cells, SynNotch gated CAR-T cells recognizing GD2 ganglioside as the gate and B7H3, i.e., CD276, as the target (Figure 4), were assayed against neuroblastoma in vitro and in xenograft mice demonstrating the specificity and efficacy of the therapy [128].

Moreover, *O*-acetylated GD2, GM3(Neu5Gc), and the globoside stage specific embryonic antigen (SSEA)-4, given their enhanced specificity for tumour cells as above discussed, have been suggested as a promising targets for CAR-T cells [117,129].

## 5. Perspectives and Concluding Remarks

Sphingolipids, S1P, and Cer play a pivotal role in different aspects related to immune response. Thus, it is not surprising that they can be involved in interactions among cancer and immune cells, at different levels. Aberrant sphingolipids and the altered release of S1P and/or Cer could alter the functionality of immune cells populating the tumour microenvironment, including Tregs lymphocytes and macrophages, and could cause immune tolerance. Thus, the manipulation of sphingolipids and S1P metabolism can be relevant to improve the efficacy of immunotherapies. SK1 knockout in mice has been demonstrated to enhance the efficacy of checkpoint inhibitors [130], whereas high SK1 expression in melanoma cells was related to reduced efficacy of anti-programmed cell death (PD)-1 immunotherapy in patients [131]. Instead, α-galactosylceramide is a promising activator of NKT cells [132].

Moreover, sphingolipids, particularly gangliosides, appeared to be ideal antigens marking indelibly tumour cells and making them recognized by immune cells effectors. The first evidence that cancer cells manipulate sphingolipid metabolism is old, at least from early ‘70s. This metabolic dysfunction leads to peculiar quantitative and qualitative differences between cancer cell sphingolipid profile and that expressed by healthy cells. Thus, the possibility of employing these molecules, particularly gangliosides, as tumour-associated antigens against which elicit the immune response soon emerged. Currently, the establishment of sensitive technologies, such as mass spectrometry, able to better investigate the chemical structures of sphingolipids allowed to identify novel forms, which can better help to target cancer cells and avoid possible toxicity events. Racotumomab constitutes an example of this type of application, but also *O*-acetylated forms could be considered to this end. In addition, the capability to synthesize artificial glycosphingolipids, manipulating their oligosaccharide chains, appeared to be a viable option to improve adaptive immune response [133]. In parallel, monoclonal antibodies and their variants are applied in different malignancies with a quite good efficacy.

Today, the most intriguing application of sphingolipid knowledge in immunotherapy could be related to CAR-T cell therapy. This is an innovative and original strategy, which deeply modified the prognosis of aggressive B-cell malignancies and appeared to totally eradicate the tumours. Unfortunately, the same success has not been achieved in solid tumours; severe side effects, low efficacy due to antigen escape and low tumour antigens specificity, low CAR-T survival, and migration into the tumour microenvironment impaired this type of application, even if many trials have been started. Gangliosides, in particular GD2, are being investigated for CAR-T cells therapy against childhood tumours, but could be explored also for other tumours such as melanomas or glioblastomas, in which similar alterations are present.

In conclusion, immunotherapy is now considered a chief strategy to treat metastatic or resistant tumours. Sphingolipids and S1P manipulation could be considered to increase its efficacy and to amply its application, acting at different levels.

## Figures and Tables

**Table 1:** Structures of sphingosine 1-phosphate, ceramide, and neutral and sialylated glycosphingolipids referred to in the text.

Molecule	Structure
Sphingosine 1-phosphate (S1P)	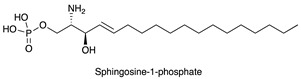
Ceramide (Cer)	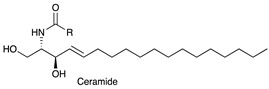
*N*-Acetylneuraminic acid (Neu5Ac)	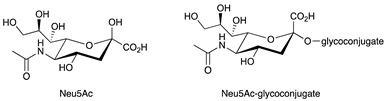
*N*-glycolylneuraminic acid (Neu5Gc)	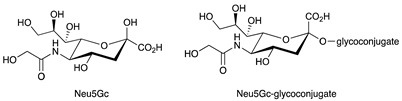
αGalactosylceramide (αGalCer)	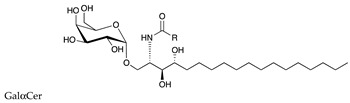
Lactosylceramide (LacCer)	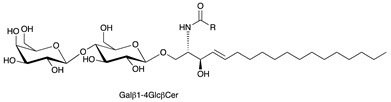
GM3 (Neu5Ac)	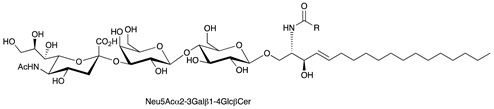
GD3	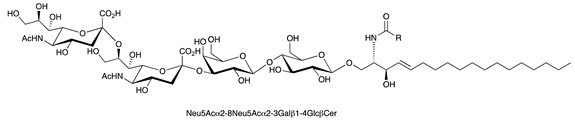
GM2	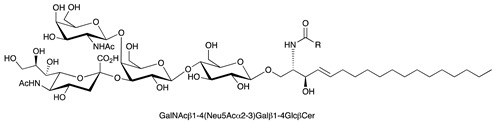
GD2	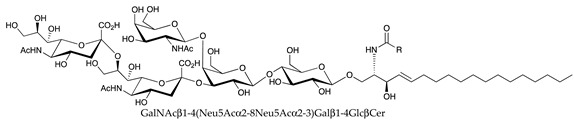
GM1	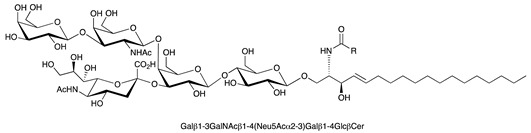
FucosylGM1	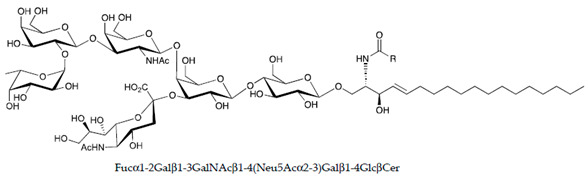
Globo-H	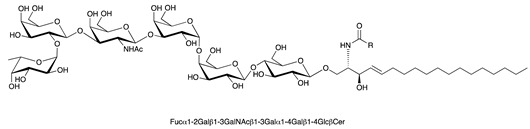

## Data Availability

Not applicable.
